# Poly(3-hydroxybutyrate)-Based Biomimetic Materials Encapsulated with Amide Derivatives of Chlorin-e6 for Advanced Photodynamic Therapy

**DOI:** 10.3390/nano16110658

**Published:** 2026-05-24

**Authors:** Polina M. Tyubaeva, Ivetta A. Varyan, Roman R. Romanov, Nikita G. Yabbarov, Maria B. Sokol, Maria R. Mollaeva, Margarita V. Chirkina, Bekzod B. Khaydarov, Evgeny A. Kolesnikov, Anton E. Egorov, Alexey A. Kostyukov, Vladimir A. Kuzmin, Olga A. Gruznova, Dmitry V. Gruznov, Ekaterina N. Shuteeva, Ekaterina A. Larkina, Elena D. Nikolskaya

**Affiliations:** 1 Department of Biological and Chemical Physics of Polymers, Emanuel Institute of Biochemical Physics, Russian Academy of Sciences, 4 Kosygina Street, 119334 Moscow, Russia; polina-tyubaeva@yandex.ru (P.M.T.); elenanikolskaja@gmail.com (E.D.N.); 2 Center for Collective Use «Scientific Equipment», Plekhanov Russian University of Economics, 36 Stremyanny Lane, 117997 Moscow, Russia; otmetkin@mail.ru; 3 Department of Functional Nanosystems and High-Temperature Materials, National University of Science and Technology (MISIS), Leninsky Prospekt, 4-1, 119049 Moscow, Russia; 4 Department of Functional Nanosystems and High-Temperature Materials, National Research Nuclear University «MEPhI», 31 Kashirskoe Highway, 115409 Moscow, Russia; 5 Laboratory of Liquid-Phase Oxidation, N.N. Semenov Federal Research Center for Chemical Physics, Russian Academy of Sciences, 4 Kosygina Street, 119334 Moscow, Russia; 6 Laboratory of Veterinary Sanitation, All-Russian Research Institute of Veterinary Sanitation, Hygiene and Ecology—Branch of Federal State Budget Scientific Institution “Federal Scientific Center—K.I. Skryabin, Ya.R. Kovalenko All-Russian Research Institute of Experimental Veterinary Medicine, Russian Academy of Sciences”, 5 Zvenigorodskoye Highway, 123022 Moscow, Russia; ekashyt@mail.ru; 7 Laboratory of Veterinary Sanitation and Environmental Safety in Beekeeping, All-Russian Research Institute of Veterinary Sanitation, Hygiene and Ecology—Branch of Federal State Budget Scientific Institution “Federal Scientific Center—K.I. Skryabin, Ya.R. Kovalenko All-Russian Research Institute of Experimental Veterinary Medicine, Russian Academy of Sciences”, 5 Zvenigorodskoye Highway, 123022 Moscow, Russia; 8 Department of Chemistry and Technology of Biologically Active Compounds, Medicinal and Organic Chemistry, Institute of Fine Chemical Technology, MIREA-Russian Technological University, Vernadskogo pr. 78, 119454 Moscow, Russia; side-line@yandex.ru

**Keywords:** poly-3-hydroxybutyrate, chlorin-e6, antimicrobial activity, PDT, electrospinning

## Abstract

In the present research, a new type of biomimetic material loaded with chlorophyll derivatives (CpDs) for photodynamic therapy based on poly(3-hydroxybutyrate) (PHB) was fabricated by the electrospinning method. Such matrices showed great potential for the advanced delivery of photodynamic therapeutic reagents to targeted regions and options for prolonged local application. The key morphological characteristics of fibrous materials were investigated. It was found that incorporation of CpDs leads to a change in the average fiber diameter from 3.5 µm to 2.1 µm, increasing porosity from 80% to 90% and accompanied by an over 3-fold increased proportion of open pores. Moreover, the CpD application facilitated fine hydrophilicity tuning, allowing an increase of this parameter up to 10% under different conditions, neutralizing the hydrophobic nature of the matrix polymer and photosensitizer. Moreover, changes in physical properties, supramolecular structure, photosensitizing effect, and singlet oxygen generation were investigated. The data obtained show that the proposed materials are great examples of convenient and reliable carriers for advanced PDT. The results obtained demonstrate high antimicrobial activity in the presence of irradiation as well as noticeable efficacy against carcinoma, both light and dark.

## 1. Introduction

Photodynamic therapy (PDT) is a method of destroying pathological tissues using light and a photosensitizer (PS), which, when activated by a certain wavelength, generates reactive oxygen species, causing cell death [1]. Among various advantages, PDT represents an organ-preserving therapy, offering a non-invasive, painless alternative to conventional cancer treatment. PDT is applied in the treatment of various forms of carcinoma, particularly in skin carcinoma, where local application has proven to be remarkably effective in minimizing tumor burden and preserving healthy tissue integrity [2,3]. PDT also demonstrates high efficacy in antimicrobial therapy, gynecology, dermatology, and cosmetology [4]. The widespread use of this method is associated with the development of a new, more effective PS. Key criteria for an optimal PS are low light/dark phototoxicity at therapeutic doses, storage stability, good luminescence (for reliable tumor diagnosis) and aqueous solubility [5]. One of the most promising PSs is chlorin-e6 and its chemical analogies [6]. Chlorin-e6 is obtained from the anaerobic alkaline hydrolysis of pheophorbide-a, and it is highly soluble in biological media [7]. Noteworthy for its low toxicity, straightforward and relatively simple synthesis, and remarkable affinity for malignant tissues, chlorin-e6 is an outstanding example of a PS for targeted cancer and antibacterial therapy [8,9,10,11].

Chlorophyll derivatives (CpDs) have also gained significant interest due to various advantageous properties. Unlike chlorin-e6, CpDs exhibit potent toxicity against cancerous cells while demonstrating complete biocompatibility with healthy tissues, emphasizing its nature as a safer alternative for clinical applications [12,13]. Moreover, CpDs’ optimal absorption wavelength range is 650–670 nm, which ensures deeper tissue penetration, overcoming a critical barrier in treating tumors located relatively deep.

Currently, there are many studies dedicated to creation carriers and nanoplatforms for CpD targeting [14,15,16,17,18]. Polyethylene glycol, poly(lactic-co-glycolic) acid, polyhydroxyalkanoates, chitosan, and a number of natural polymers are widely used for advanced CpD delivery [19,20,21,22,23,24]. Table 1 sums up the state-of-the-art polymer-based PDT systems.

Most of the polymers are characterized with low glass transition temperature (lower than the subfibrile temperature), resistance to hydrolysis, and low photostability [41]. Thus, the challenge persists in identifying effective carrier systems that are able to safely and effectively transport CpDs to target tissues, maximizing treatment efficacy and minimizing adverse effects.

Great attention should be paid to the polyhydroxyalkanoates, especially to polyhydroxybutyrate (PHB) due to its low glass transition temperature from −10 to 9 °C, high melting points of 140–180 °C, and resistance to hydrolysis, which do not affect the rate of biodegradation in vitro and in vivo, and high biocompatibility [42]. PHB degradation does not lead to micro-environment acidification, like in poly(lactic-co-glycolic) case [43]. Moreover, PHB is a semicrystalline isotactic stereo regular polymer with 100% R configuration, ensuring high degradation properties in vivo [44]. PHB is suitable in the fabrication of nanostructures and scaffolds with a developed surface area, which are effective in controlled drug release [45,46,47,48,49]. PHB is applied broadly in tissue engineering [50]. Several studies showed the possibility to control the porosity, mechanical properties and hydrophilicity of PHB-based scaffolds, allowing prediction of the release rate of PS [51,52]. Other studies reported PHB’s effective crystallization control in the presence of various additives, some of them acting as nucleating particles in the process of fiber formation, which influences cell proliferation and biodegradation [53,54]. Existing PHB-PS systems demonstrate efficiency [55,56]. However, the efficiency of the photosensitizer can be increased by synthesizing the most optimal analog, taking into account the tasks of the mutual influence of the polymer and photosensitizer to obtain a controlled and stable mobile system for PDT.

This study is dedicated to the development of flexible polymeric materials as a carrier for CpDs. Thus, the main purpose of this study was to design, formulate and evaluate properties of PHB-CpD electrospun matrices. In this study, the potential of PHB-CpD electrsopun matrices for PDT in human epidermoid carcinoma treatment and antibacterial treatment was explored in vitro.

## 2. Materials and Methods

### 2.1. Materials

#### 2.1.1. Polymer

Biopolymer poly-3-hydroxybutyrate (PHB) was obtained through microbiological synthesis (16F series, produced by BIOMER, Frankfurt, Germany), characterized by a crystallinity of 59%, a molecular weight of 435 kDa, and a density of 1.248 g/cm^3^.

#### 2.1.2. Photosensitizers

**Scheme 1:**
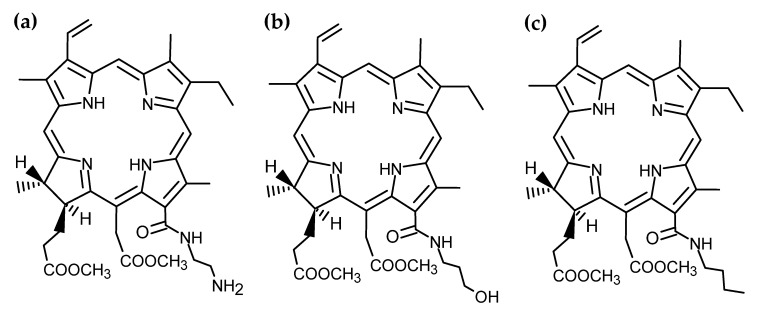
Structural formulas of mC_2_H_4_NH_2_ (**a**), mC_3_H_6_OH (**b**), and mC_4_H_10_ (**c**).

All photosensitizers were obtained in powdered form by organic synthesis from methyl pheophorbide a according to the known method of opening of the extra ring in pheophorbide a by the action of primary amines. Amide derivatives of chlorin-e6 were synthesized and characterized according to previously reported procedures. The synthesis of 13(1)-N-(2-aminoethyl)amide-15(2),17(3)-dimethyl ester of chlorin-e6 (mC_2_H_4_NH_2_, Scheme 1a) was carried out in compliance with the method in [57], 13(1)-N-(3-hydroxypropyl)amide-15(2),17(3)-dimethyl ester of chlorin-e6 (mC_3_H_6_OH, Scheme 1b) was synthesized according to [58], and 3(1)-N-butylamide-15(2),17(3)-dimethyl ester of chlorin-e6 (mC_4_H_10_, Scheme 1c) was synthesized according to [59].

The UV–Vis spectra of all chlorin-e6 derivatives are similar, correspond to the spectra presented in the articles [57,58,59] and retain the characteristics of chlorin-e6, since incorporation of functional groups far from the chlorin ring does not affect the conjugated electronic system of the latter.

#### 2.1.3. Reagents

Chloroform (CL), indocyanine green (ICG), Tween 80, dimethyl sulphoxide (DMSO) and phosphate-buffered saline (PBS) were purchased from Amresco (Solon, OH, USA). Trypsin and fetal bovine serum (FBS) were purchased from Hyclone (Logan, UT, USA). Ethylenediaminetetraacetic acid (EDTA) was purchased from Serva (Heidelberg, Germany). DMEM culture medium was purchased from Gibco (Waltham, MA, USA). 3-(4,5-dimethyl-thiazol-2yl)-2,5-diphenyltetrazoliumbromide (MTT) was purchased from Sigma-Aldrich (St. Louis, MO, USA). Water was purified in a Millipore Milli-Q Plus system form Merck (Darmstadt, Germany).

### 2.2. Electrospinning

Biomimetic matrices based on PHB were obtained by the electrospinning method (ES) using an EFV-1 single-capillary lab scale unit (Moscow, Russia). ES solutions of PHB and PHB-CpDs were prepared by the dissolution of 7 wt. % of PHB in CL under a vigorous stirring at 60 °C for 10 min until the solution was homogenized in an automatic stirrer. CpDs were dissolved in CL at 25 °C for 10 min until the solution was homogenized and then they were injected into a polymer solution. CpD content was 0.3 wt. %. The conditions of the ES were the following: humidity was 35%; diameter of the inner needle was 0.1 mm; duration was 4 h; the distance between a spinneret and a collector was 200 mm; stationary collector area was 30 mm^2^; flow rate was: 6.6 mL/min for PHB, mC_2_NH_2_ and mC_3_OH and was 6.4 mL/min for mC_4_; voltage was ±17–18 kV; ES was performed at room temperature. The viscosity of ES solutions was controlled with Brookfield Rotary Viscometers DV2TLV. Electrical conductivity of the ES solutions was controlled with SanXin DDS-11C Laboratory Conductometer, with a measurement error ±1.5%.

### 2.3. Methods

#### 2.3.1. Characterization of PHB-CpD Matrices

Morphology of the electrospun samples was studied with Scanning Electron Microscopy (SEM) using a Tescan VEGA3 scanning electron microscope (Tescan, Brno, Czech Republic) [60]. All test samples 10 × 10 mm^2^ were covered with a platinum layer. The SEM micrographs were analyzed using the Femto Scan Online software.

The elemental mapping for determining CpD distribution in the PHB matrix was studied by Energy-Dispersive X-ray Spectroscopy (EDS) using a Tescan VEGA3 energy-dispersive X-ray unit. The measurements were performed for the platinum-coated samples of 10 × 10 mm^2^.

Surface density of the PHB-CpD samples was evaluated gravimetrically using a Balance XPR106DUHQ/A analytical weighing-machine (Mettler Toledo, Columbus, OH, USA) [61]. Samples were 10 × 10 mm. The average value was estimated from 10 measurements. Experimental error was below 3–5%.

Permeability to air was measured according to ISO 5636-5 using a Gurley densometer [62]. The test area of the samples was 6.5 cm^2^, pressure was 1.22 MPa, and test volume was 100 mL. Relative experimental error was ±5%.

Wettability was measured by 5 drops in 3 areas of each sample on an FMA050 optical microscope (Saint Petersburg, Russia) with Altami studio 3.4 Software [61]. Water droplet volume was 2 μL. Test samples were 30 × 30 mm^2^. Relative experimental error was ±0.8%.

Mechanical properties were studied using a DVT GP UG compression testing machine (Devotrans, Istanbul, Turkey) [63]. The strain rate speed was 5 mm/min (without preloading). Testing conditions, according to ISO 139:1973 “Textiles—Standard atmospheres for conditioning and testing” were +21 ± 1 °C, RH 60%, and 760 mm Hg. Sample preparation was in accordance with ISO 9073-3 “Nonwovens—Test methods—Part 3: Determination of tensile strength and elongation at break using the strip method”. Test samples were 10 mm (width) and 50 mm (length). Thickness of the samples was controlled in 5 points and was 0.27–0.3 mm. Tensile strength, elongation at break, and Young’s module were calculated automatically. The average values were calculated from 10 measurements with the standard deviation. One-way ANOVA (Bonferroni correction) in comparison with PHB matrices as a control group was used for determination of the *p*-value.

Differential Scanning Calorimetry (DSC) measurements were made using the Netzsch 214 Polyma (Netzsch, Selb, Germany) in an argon atmosphere [64]. The heating and cooling rate was 10 °K/min. Test samples were cut from 3 different areas of the electrospun material with a total weight of 7 mg. The enthalpy of melting and melting temperatures were calculated using Netzsch Proteus software.

Fourier Transform IR-Spectroscopy (FTIR) measurements were made using a Bruker LUMOS II Research Infrared Fourier Microscope (Bruker, Karlsruhe, Germany) with the module for measuring multiple disturbed total internal reflection on diamond crystal [65]. The range of the measurement was 600–4000 cm^−1^, and the resolution was 2 cm^−1^. All spectra were taken at least 10 times at 3 areas of the sample and brought to an average value.

Raman spectra of the samples were acquired using an NTegra spectrometer (NT-MDT, Moscow, Russia) with a 532 nm excitation (4.0 mW on the sample) and 100× objective with a range of 140–2650 cm^−1^. Each sample was examined in five to eight random spots. Three spectra with a collection time of 60 s were recorded for each spot. Wavelengths were calibrated using positions of Ne emission lines [66]; Raman shifts were calculated using an excitation wavelength of 532.0 nm. All acquired spectra for each sample were averaged. For presentation purposes, the baseline was subtracted using the built-in function Polynomial baseline of the OPUS 7.0 software (Bruker Optic GmbH, Ettlingen, Germany).

The release kinetics were investigated according to the technique in [60]. Samples (20 × 40 mm) were incubated under constant shaking of 180 rpm at +37 °C over 25 days in 35 mL of release media containing 0.1 M PBS pH 7.4 and 1% (*w/v*) Tween 80. The supernatant samples were picked at predominant time intervals, and an equal volume of solvent was added to the release system to keep the volume constant during the experiment. The released mC_2_NH_2_, mC_3_OH, and mC_4_ absorbance was determined by UV spectrophotometry Helios Alpha (Thermo Spectronic, Cambridge, UK) at 403 nm. The release data are presented as the average value of three specimens with the standard deviation. The logP calculation was performed with ACD/labs v 10.0 software.

#### 2.3.2. Photochemical Assay of CpDs and PHB-CpD Matrices

Luminescence measurements were completed using the FluoTime 300 spectrometer (PicoQuant GmbH, Berlin, Germany) [67] in the IBCP RAS (125020501478-5). The probes were placed in a solid sample holder at a 45-degree angle towards the excitation source and registration. Excitation was performed using a Xenon lamp (λ_ex_ = 500 nm). Emission data were collected at 720 nm. Test samples were 10 × 10 mm^2^.

Indocyanine green (ICG) was used as a singlet oxygen detection probe. In order to diminish ICG adsorption on the surface of the polymer, Triton X-305 (6 × 10^−4^ M) was added. The polymer matrix pieces were 10 × 10 mm^2^, with incorporated compound 1, and the ones without photosensitizers (blank samples) were placed in a 1 × 1 cm glass cuvette. The irradiation was performed at a 15 cm distance with a halogen lamp (50w) using an optical glass filter (transmission > 400 nm). After certain periods of irradiation, the solution absorption spectra were recorded.

#### 2.3.3. Biological Assay of PHB-CpD Matrices

The cell line A431 (human epidermoid carcinoma) (ATCC, Manassas, VA, USA) was maintained in DMEM supplemented with 10% fetal bovine serum and gentamycin (50 µg/mL) [16]. Cells were grown in plastic 25 cm^2^ cell culture flasks at +37 °C in humidified atmosphere containing 5% CO_2_ (MCO-17AIC, Sanyo, Osaka, Japan). Cells were seeded twice per week before reaching 80% confluence by detachment with trypsin (0.05%)/EDTA (0.02%) solution.

A431 cells were seeded in 96-well plates (3000 cells per well) 24 h before the experiment and incubated under standard conditions [16]. The mC_2_NH2, mC_3_OH and mC_4_ substances were predissolved in DMSO (1 mg/mL) and added in triplet in the concentration range of 0.025–0.0000078 into wells. After 1 h of incubation of the cells in the presence of the substances, the plate was irradiated with a 660 nm light-emitting diode plate for 20 min with a 1.7 J/cm^2^ power LED and then incubated for 72 h. Cell survival was determined using a standard MTT assay [68]; 50 µL MTT in DMEM (1 mg/mL) was added into each well. After cell incubation at +37 °C, the medium was removed, and precipitated formazan crystals were dissolved in 100 µL DMSO. Following this, the absorption intensity of formazan was measured at 540 nm on a microplate reader. Cell viability was determined as the percentage of untreated controls. The responses were measured in triplicate.

To evaluate the activity of the substance-loaded PHB membranes, we prepared 1 cm (d) disks and placed them onto the bottom of 24-well plates (3 repeats). The 1 cm disks were cut off the membrane by the sterile sharpened metallic stencil in aseptic conditions in order to keep the surface area and quantity of material placed into the wells constant among different samples. Next, 10^4^ of A431 cells in 1 mL of DMEM media was seeded onto the membranes. After 24 h, the plates were irradiated with a 660 nm light-emitting diode plate for 20 min with a 1.7 J/cm^2^ power LED and then incubated for 72 h. Cell condition was fixed at 24, 48 and 72 h of incubation using a Nikon Diaphot PC microscope at 40× magnification and a Levenhuk M1400Plus camera (Prague, Czech Republic) (control plate was not exposed to irradiation at 660 nm and was incubated in the dark during 72 h) [16]. Next, the medium was removed, the precipitated formazan crystals were dissolved in 500 µL of DMSO, and then absorbance at 540 nm of each sample was measured.

To compensate for the absorption of substances in the films, materials of approximately similar area were placed in the wells of a 24-well plate and incubated in 500 μL of DMSO—the optical absorbance of the obtained solution at 540 nm was subtracted from the absorbance of the experimental samples populated with A431 cells.

The DMSO solution samples incubated with substance-loaded PHB 1 cm membranes were used for OD 540 absorbance compensation.

#### 2.3.4. Antimicrobial Assay of CpDs and PHB-CpD Matrices

The *Staphylococcus aureus* (209-P strain) culture was cultivated on a slanted MPA in a dry-air thermostat at 37 °C for 24 h. Suspensions of 10^9^ CFU/mL were prepared from daily cultures in sterile saline solution according to the turbidity standard. The 10^6^, 10^5^ and 10^4^ CFU/mL dilutions of *S. aureus* were prepared by the titration method and were inoculated into Petri dishes with sterile MPA. PHB-CpD samples with an area of 1 cm^2^ were placed in the center of the inoculated Petri dishes. A 100 µL volume of free CpDs dissolved in DMSO (dose of 30 µg) was placed in wells created with a sterile well punch. To study the photodynamic activity of the preparations, some Petri dishes with samples were irradiated at 660 nm (light dose—16.2 J/cm^2^) for 25 min using the LED lamp (Omega light, Seoul, South Korea). The lamp calibration according to the “power” and “wavelength” criteria was carried out in a metrology laboratory. The specimens intended to detect the light-independent effect were not irradiated. Then, samples were incubated in a thermostat (37 °C, 72 h). The results were taken into account daily according to the diameter of the growth inhibition zone (mm). To confirm the lack of toxicity of the polymer, the PHB samples without active substances were used. The experiments were carried out in triplicate.

Images of *S. aureus* cells incubated with irradiated and non-irradiated CpDs were obtained using scanning electron microscopy (SEM) method. A total of 0.5 mL of *S. aureus* (10^4^ CFU/mL) and 100 µL of the CpDs (30 µg) were added to a test tube with 5 mL of sterile MPB. The control samples were not exposed to preparations. Some samples were irradiated under a LED lamp for 25 min. Next, incubation was carried out for 12 h in a dry-air incubator at 37 °C. Then, 1.75 mL of each sample was placed in test tubes and centrifuged at 14,500 rpm for 15 min in a centrifuge (Eppendorf MiniSpin plus, Eppendorf, Hamburg, Germany). The supernatant liquid was removed, and 1.75 mL of sterile ddH_2_O was added to the sediment. Then, the centrifugation cycle was repeated to remove the supernatant. A total of 0.5 mL of sterile ddH_2_O was added to the resulting centrifugate and shaken on a vortex mixer (Vortex V-1 plus, BioSan, Riga, Latvia) until a homogeneous suspension was obtained. Then, 50 µL of the suspension was evenly distributed in a thin layer over the surface of the cover glass. The coverslips were placed in a sealed desiccator (DWK Life Sciences, Mainz, Germany). Fixation was carried out with vapors of 25% (*v*/*v*) glutaraldehyde (BASF AG, Ludwigshafen am Rhein, Germany) in a tightly closed desiccator at room temperature (22 °C ± 2 °C) for 15 min in a fume hood (Korsa, Moscow, Russia). Then, the samples were dehydrated in ethanol (JSC Rosspirtprom, Moscow, Russia) with increasing concentration of 30%, 50%, 70% and 96% (*v*/*v*), maintained for 10 min in each solution. After dehydration, the samples were dried in a fume hood until the solvent had completely evaporated. The cell morphology was studied using a Coxem EM-30 Plus microscope (Coxem, Daejeon, Korea). For this purpose, the fixed sample was secured to a sample holder using carbon double-sided adhesive tape and coated with a thin layer of gold using an SPT-20 ion coating system (Coxem, Daejeon, Korea) in the 100 s and 5 mA regime. The samples were scanned at 4000× magnification.

#### 2.3.5. Statistics and Data Analysis

The results were expressed as mean (±S.D.) unless otherwise specified. Statistical calculations were carried out using Design Expert 7.0 (Stat-Ease Inc., version 7.0; Design-Expert software; Minneapolis, MN, USA) Data Acquisition Station (DAS) (MREL Group of Companies, version 2.0; Data Acquisition Station, Kingston, ON, Canada). Statistical analysis was performed using a two-tailed unpaired Student’s *t*-test or ANOVA test. *p* < 0.05 was considered as significant.

## 3. Results and Discussion

### 3.1. Electrospinning of PHB-CpD Matrices

It is important to establish precisely the localization of the photosensitizer as well as to control the concentration, due to the limited operation range of porphyrins [69]. Photosensitizer molecules are highly hydrophobic, which hinders the penetration through the cell membrane; thus, it is important to control the hydrophilicity of the photosensitizer-containing material. It is also important to note that the amphiphilicity of the compound is of great importance, since overly hydrophobic materials will pass well into the membrane by passive diffusion but poorly dissolve in the body media, and overly hydrophilic materials will dissolve well in body media but face challenges overcoming the lipid bilayer of the plasma membrane by passive diffusion [70]. Increased drug import into the cancer cell can stimulate resistance, which emphasizes the importance of developing photosensitizer carriers with low concentrations of the active substance and the possibility of controlled release [71]. The formulation of fibrous biomimetic materials by ES is an effective approach to solve the problem, as it allows guarantee of a uniform additive distribution even at low concentrations in the polymeric matrix structure, the control of many material properties, and productive and hardware-intuitive results [72].

**Figure 1:**
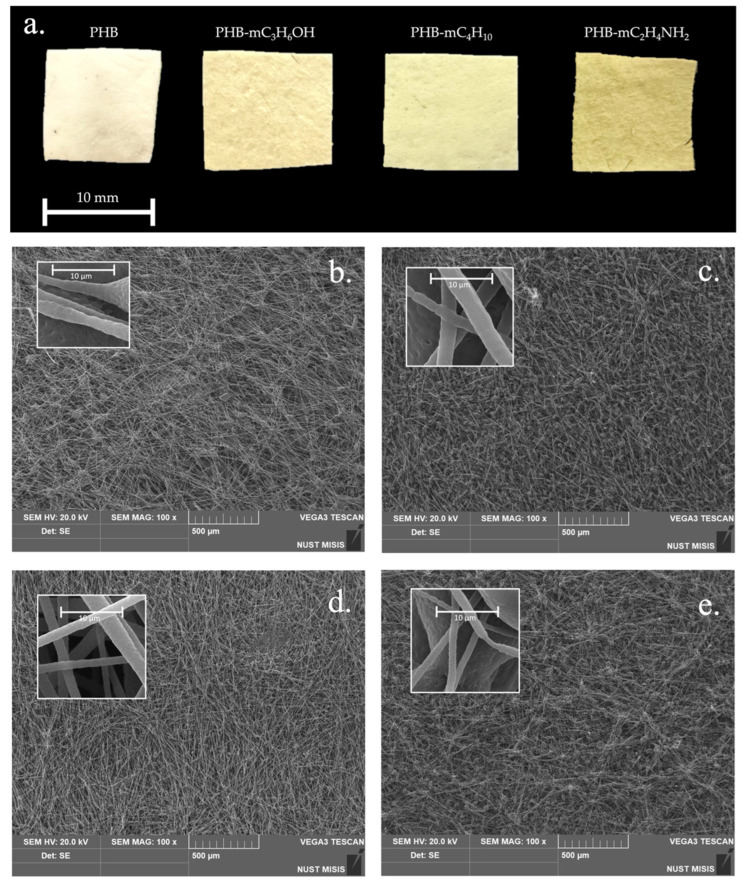
Photographs (**a**) of obtained materials (bar, 10 mm) and SEM images of PHB (**b**), PHB-mC_3_H6OH (**c**), PHB-mC_4_H_10_ (**d**), and PHB-mC_2_H_4_NH_2_ (**e**) electrospun matrices (bar, 500 μm).

Based on the results of previous work [73], an optimum concentration for PHB suitable for ES was determined as 7% (*w*/*v*); the amount of PS was 0.3% (*w*/*v*). CL was used as a solvent, since both the CpDs and the polymer have good solubility [74]. A range of 200–500 kDa PHB was used due to the possibility of this molecular weight to ensure the material rapid biodegradation in vivo—within 10–15 days in the case of non-woven fabrics [60,75]. Earlier [76], it was shown that 6 and 8% (*w*/*v*) PHB solutions are characterized by a biased balance of viscosity and electrical conductivity, preventing the production of a highly developed fibrous structure with an average fiber diameter less than 4 µm. However, CL application causes defects in fiber formation—thickenings, which are often found in electrospun nonwoven PHB materials [77,78,79]. Usually, researchers solve this problem by changing the solvent or using a combination of solvents, including CL, dichloromethane, dimethylformaldehyde [80], and plasticizers, modifying additives, or extra polymer introduction, which increases electrical conductivity [81]. In our case, the use of a modifying additive was investigated, which had a significant effect on the ES properties of the polymeric solution. The ES conditions of PHB-CpD matrix fabrication are shown in Table 2. Photographs of the obtained materials are shown in Figure 1a.

Thus, CpDs had a significant effect on the ES properties, shifting the balance of viscosity and electrical conductivity; unfortunately, remaining defects in the fibers were observed. Thus, a 40% increase in the electrical conductivity of the PHB-CpD solutions turned out to be significant and was expressed in a noticeable decrease in the average diameters. At the same time, a 20% increase in viscosity turned out to be almost imperceptible in the polymer-PS system. These viscosity changes probably found expression in the formation of local thickenings due to the lack of additional solutions for optimizing electrospinning parameters. The results obtained push the prospects for further research in optimization of the PHB-CpD matrix ES process.

### 3.2. Characterization of Electrospun PHB-CpD Matrices

The morphology of electrospun PHB-CpD matrices was analyzed by SEM and is shown in Figure 1. The structure of nonwoven material in all cases could be characterized as a highly developed fibrous structure in all cases.

**Figure 2:**
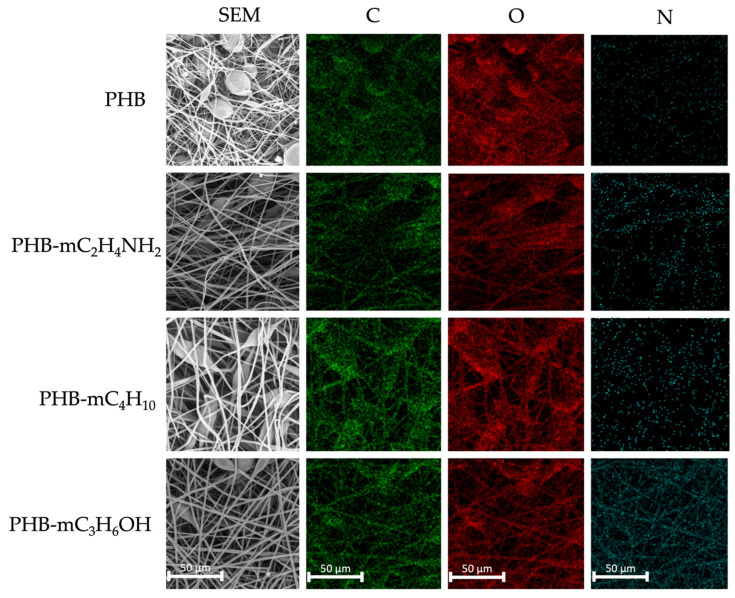
The elemental mapping of PHB-CpD electrospun matrices (bar, 50 μm).

The elemental mapping for determining CpD distribution in the PHB matrix was studied by the EDX method (Figure 2). The distribution of the nitrogen atom (N, blue color) should be considered the most significant, since it is characteristic of CpDs as a part of a heterocycle. Despite small concentrations of CpDs, a fairly uniform increase in the proportion of nitrogen atoms on the sample surface is observed. Predominantly, the color density is combined with the morphology of the sample; however, PHB-mC_4_H_10_ is characterized by the lowest particle density at the surface, which corresponds with the profile of delayed release.

The implementation of CpDs led to a reduction in average fiber diameters, from 3.5 µm to 2.1 µm for PHB-mC_3_H_6_OH, 2.6 µm for PHB- mC_4_H_10_, and 2.2 µm for PHB-mC_2_H_4_NH_2_. It is important to note that the additive reduced the distribution of average diameters, allowing better control of the ES efficiency. These changes could be attributed to the altered viscosity and electrical conductivity (Table 2) of the PHB-CpD solutions due to the introduction of more electrically conductive particles. It is likely that the proposed CpDs increase electrical conductivity due to the structure of the radical, which is found in many polar molecules used to optimize the ES process [82]. However, it should be noted that concentration of 0.3% may not be sufficient to fully optimize the ES process, since thickenings are still found on the surface of the fibers.

Additionally, density measurements showed that the bulk density of the materials decreased from 0.3 g/cm^3^ to 0.16 g/cm^3^ for both PHB-mC_3_H_6_OH and PHB-mC_4_H_10_, and to 0.18 g/cm^3^ for PHB-mC_2_H_4_NH_2_. The fraction of open pores was estimated according to the Gurley method by analyzing the volume of air passing through the material per unit of time [61]. The air permeability of PHB-CpD matrices significantly increased, from 0.38 mL to 4.35 mL for PHB-mC_3_H_6_OH, 2.35 mL for PHB-mC4H10, and 3.75 mL for PHB-mC_2_H_4_NH_2_.

Heightened permeability was linked to the absence of interfiber adhesions, often seen when solvent evaporation is incomplete at the stage of laying fibers on the precipitation electrode, as observed in the pure PHB case [83]. The addition of polar molecules may significantly affect ES and provide much higher molding properties of the material even at lower concentrations [84]. The role of polar molecules was consistent with the wetting angle variations, which characterizes surface hydrophilicity. It is important to note that both PHB and PS are intrinsically hydrophobic [85]. Nevertheless, the application of polar molecules markedly improved ES properties, even at lower concentrations, according to the wetting angle evaluation results: angles decreased from 129° to 120° for PHB-mC_3_H_6_OH and to 126° for PHB-mC_2_H_4_NH_2_, and they increased to 130° for PHB- mC_4_H_10_, demonstrating the significant influence of the radicals’ hydrophilic groups –OH and –NH_2_ on the surface properties. In contrast, the addition of mC_4_H_10_ led to the increase of the hydrophobicity of PHB. Notably, these wetting angle changes were primarily chemical, as they do not align with variations in fiber diameters or material porosity.

### 3.3. Thermal Properties of PHB-CpD Matrices

**Figure 3:**
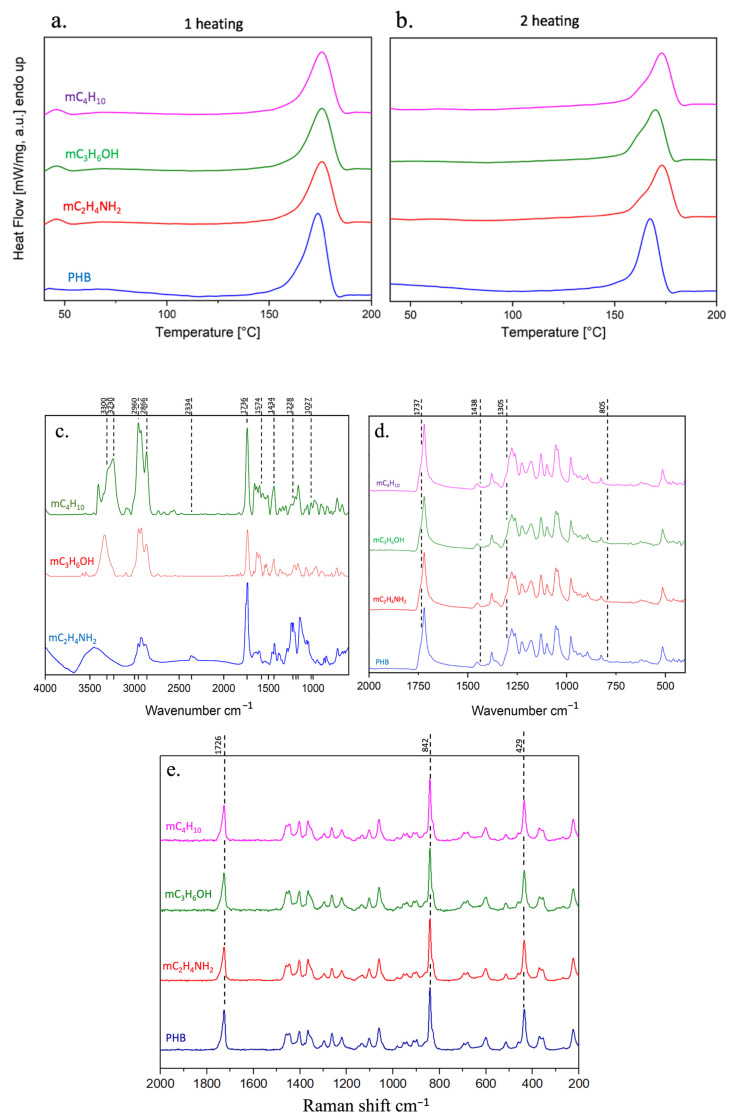
DSC curves of PHB-CpD electrospun materials: first heating cycle (**a**) and second heating cycle (**b**); FTIR spectra of pure CpDs (**c**) and PHB-CpD electrospun materials (**d**); Raman spectra of PHB-CpD electrospun materials (**e**).

PHB is a semicrystalline polymer with tendency to cold crystallization and the formation of spherulites [86,87]. Pure PHB crystallizes very slowly and has an extremely low density of crystal-nucleating agent [88]. Therefore, various nucleating particles are often used to accelerate the crystallization process. The introduction of nucleating particles and additives of various natures ensures their localization in the amorphous phase of the polymer, often at the crystallite boundary, which can affect the overall degree of crystallinity. The DSC study showed that the introduction of CpDs has a similar effect on the crystallization of PHB. The results of the study of the thermal properties of PHB-CpD matrices and the crystalline phase are shown in Table 3. In all cases of introducing CpDs to PHB the melting temperature rises slightly, and the crystallinity degree decreases by 12–17% for the first heating. It is assumed that a higher melting point during first test corresponded to more regular, large, but less dense crystalline structures, stimulated by CpD presence, while general content of crystalline phase was decreased. However, the second heating test provided additional data characterized with reduced contribution of the ES process due to the first melting. The degree of PHB crystallinity during the first and second tests varied about 10% indicating the presence of small crystalline defects, which were not able to recrystallize again under the DSC experiment conditions. However, this difference was not observed in PHB-CpD matrices, where the decrease in the PHB crystallinity did not exceed 4%. Similar changes were observed during melting point analysis. Moreover, some peculiarities in the shapes of the melting peaks were noticed (Figure 3): the major first heating (Figure 3a) peaks of all samples had a symmetrical appearance; the CpDs caused a slight endothermic effect at 40–50 °C; but, during the second heating (Figure 3b) an appearance of pronounced and noticeable low-temperature 150–160 °C indicating the fine crystalline fraction release was observed.

### 3.4. FTIR of CpDs and PHB-CpD Matrices

Figure 3c,d shows the FTIR spectra of pure CpDs and PHB-CpD electrospun materials. Special attention was paid to the CpD-characteristic bands: hydrogenated pyrrole ring IV appeared at 1027, 1164, 1191, 1228, and 1242 cm^−1^ [89]; methine bridges appeared in the high-frequency region of 1574–1644 cm^−1^ [90]; the aromatic ring bands appeared at 1305–1440 and at 1600 cm^−1^; C=O at 1737 cm^−1^; planar deformation of tetrapyrrole at 750–805 cm^−1^. The expected similarity of spectra is observed in the 1736 cm^−1^ (–C=O), 2866 cm^−1^ (valence vibrations of –CH) and 1434 cm^−1^ (–CH_2_) bands. Despite the general similarity of the CpD spectra, there are noticeable differences in the intensity of the bands (Figure 3) in the area of oscillation of the -OH groups (3200–3600 cm^−1^), in the area of fluctuations of the C–H bond in the radical chain (2960 cm^−1^), in the field of multiple bond fluctuations (2334 cm^−1^), and in the area of the –NH signal (1550–1650 cm^−1^). However, none of the CpD bands are observed on the obtained spectra of the PHB-CpD systems. However, all these bands could partially overlap with the bands of PHB. FTIR data comparison among samples and the spectra library (OPUS, Bruker) revealed a lack of pronounced differences—all spectra looked relatively identical and were similar to pure PHB. Thus, the absence of a pronounced chemical interaction between PHB and CpDs was observed. This phenomenon can be partially explained by the inconsistency of CpD distribution within the fiber—the FTIR spectra generally reflect an upper-layer amorphous molecular structure where CpDs are almost absent.

Raman spectra of the PHB-CpD electrospun materials are shown in Figure 3e (2000–200 cm^−1^). The Raman spectrum of the PHB shows intense bands at 1726, 840 and 434 cm^−1^ that correspond to C=O, C–O–C, and–CH_2_, respectively. The spectrum is consistent with other reports [91]. The standard deviation of the 840 cm^−1^ band position between individual spectra of the same sample varied from 0.4 to 0.9 cm^−1^. No band shift exceeding the standard deviation of the measurement was detected during comparison of spectra of pure PHB and PHB-CpDs.

### 3.5. Mechanical Properties of PHB-CpD Matrices

**Figure 4:**
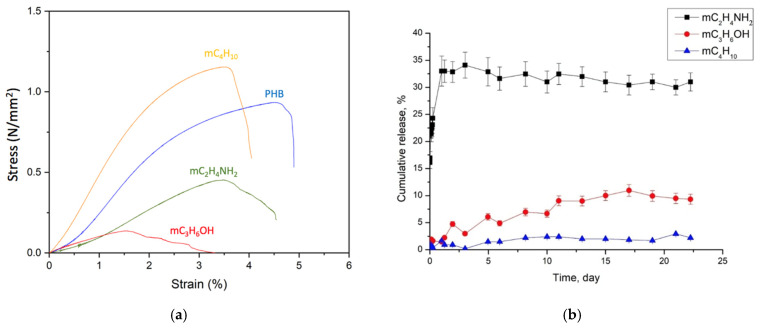
Stress–strain curves of PHB-CpD matrices (**a**); in vitro, kinetic profiles of CpD release from electrospun matrices in 0.01M PBS pH 7.4 and 1% (*w*/*v*) Tween 80 at 37 °C (**b**).

Table 4 shows the mechanical properties of the PHB-CpD matrices, and Figure 4 displays the average stress–strain curves of the electrospun matrices. It is evident that all samples exhibited increased brittleness and decreased elongation, except PHB-mC_4_H_10_. The DSC method showed that incorporating CpDs into ES solutions reduces the crystallinity of PHB by 12–17% and raises the melting temperature by 2–4 °C [74]. PHB, typically a semicrystalline and brittle polymer, influences the mechanical properties appropriately. Changes in the supramolecular structure, a decreased average diameter and reduced number of mutual meshes, as well as the presence of glued fibers collectively led to the observed changes in strength properties. However, the elastic modulus of the fibers, due to the fibrous structure, suggests that they are highly flexible [92].

### 3.6. Release of CpDs from PHB-CpD Matrices

Figure 4b shows in vitro release of CpDs from electrospun PHB-CpD systems. The issue of high hydrophobicity of PHB-CpDs was reduced by the TWEEN80 1% (*w*/*v*) application according to the standard technique.

After the first day, a burst release of up to 24% for PHB-mC_2_H_4_NH_2_ was observed (Figure 4). Next a slow-release phase occurred, releasing about 32% over the subsequent 22 days. Notably, PHB-mC_4_H_10_ and PHB-mC_3_H_6_OH did not exhibit this initial burst release. Instead, a sustained release was demonstrated, with PHB-mC_3_H_6_OH releasing up to 2% and PHB-mC_4_H_10_ up to 9% over 22 days.

The hydrolysis rate of the polymer matrix significantly influences the drug release [93]. Thus, PHB’s high hydrophobicity could contribute to prolonged drug release in aqueous media due to slow hydrolysis [94]. It is known that a number of factors influence the rate of substance release: the type of polymer matrix, the nature of the medium and the type of substance. Olkhov et al. reported that the crystallinity of PHB film affects burst release and fiber hydrolysis: higher crystallinity correlates with a lower hydrolysis rate and reduced burst release [82].

The proportion of open pores of materials changes as follows—PHB-mC_4_H_10_ < PHB-mC_2_H_4_NH_2_ < PHB-mC_3_H_6_OH—while the average diameters varied as follows—PHB-mC_3_H_6_OH < PHB-mC_2_H_4_NH_2_ < PHB-mC_4_H_10_. PHB-mC_4_H_10_ and PHB-mC_2_H_4_NH_2_ displayed the same wetting angle (130°), while PHB-mC_3_H_6_OH showed 120°. Crystallinity varied as follows: PHB-mC_3_H_6_OH < PHB-mC_4_H_10_ < PHB-mC_2_H_4_NH_2_. Thus, none of these factors correlated with the release results. In addition, the nature of the surface micro-landscape often relates directly the hydrophobicity, mitigating the media access to the material surface and the substance escape from the polymeric matrix. Figure 4 shows the surface of different PHB-CpD systems. It is clearly seen that there is an absence of a direct relationship between the depth and topology of the landscape on the surface of the fibers and the kinetics of the CpD release.

The observed burst release in mC_2_H_4_NH most probably occurred due to rapid diffusion transport from the film to the hydrated interfibrillar space [82].

Presumably, the absence of burst release in mC_3_H_6_OH and mC_4_H_10_, compared to mC_2_H_4_NH_2_, can be attributed to differences in their structure and physical properties. Hydrophobicity comparison using logP values showed that mC_2_H_4_NH_2_ (logP 5.68) is more hydrophilic than mC_3_H_6_OH (logP 6.07) and mC_4_H_10_ (logP 8.07), influencing the drug release rate. Previously, it was shown that even small differences in logP influenced the drug release rate. Sustained release from PHB fibers as observed in mC_2_H_4_NH_2_, mC_3_H_6_OH, and mC_4_H_10_ is advantageous for biomedical applications.

This feature could affect the degree of the development of the surface microstructure. Additives are able to influence the formation of the crystalline structure during the curing of the polymer-PS solution; however, the additives are located in the amorphous phase of PHB. The release occurs primarily due to hydrolysis and degradation of the amorphous phase. Despite the fact that a more developed surface can hydrolyze faster from the point of view of microrelief, a more developed surface differs in terms of greater disequilibrium, which may make it difficult for the additive to exit. We observe this for the PHB-CpD system. Thus, these factors compensate for each other and make it most difficult for mC_4_H_10_ to release.

### 3.7. Luminescence Spectroscopy of PHB-CpD Matrices

**Figure 5:**
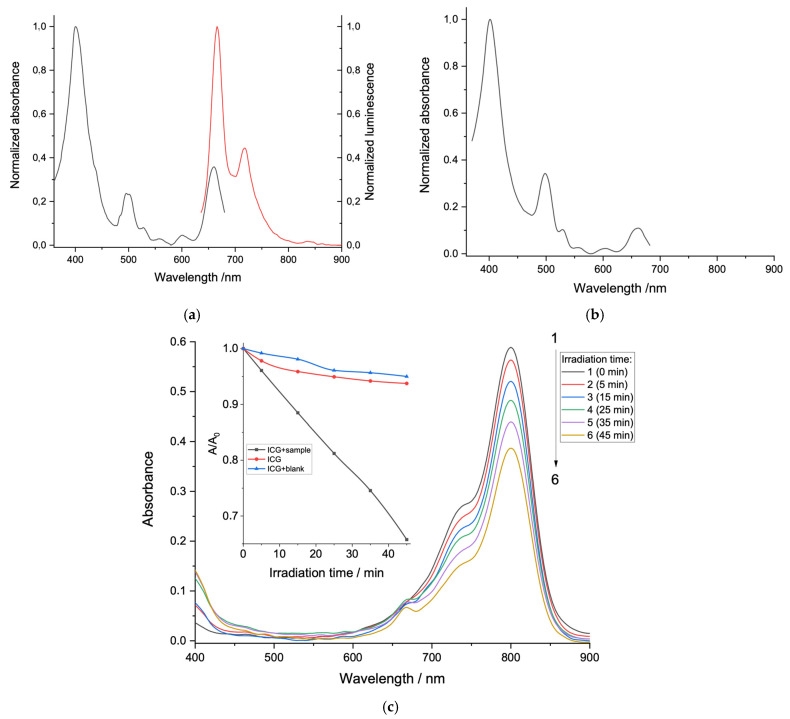
Normalized luminescence excitation (black, λ_reg_ = 720 nm) and luminescence emission (red, λ_ex_ = 500 nm) spectra for the PHB-mC_4_H_10 4_ matrix (**a**); normalized luminescence excitation (black, λ_reg_ = 720 nm) for mC_4_H_10_ (**b**); absorption spectra of ICG (2.3 × 10^−6^ M) in water in the presence of the PHB-mC_4_H_10_ matrix at different irradiation times (broadband white light > 400 nm, 20 mW/cm^2^). Inset shows the absorbance change at 800 nm at different irradiation times (**c**).

Luminescence spectra of the PHB-based matrices with CpD molecules were characterized by two intensive absorbance bands around 670 nm and 720 nm (Figure 5). The excitation spectra aligned with common chlorin spectra, showing an S-band near 400 nm and several Q-bands (500 nm and 660 nm). Notably, all CpD samples exhibited a high degree of spectral similarity, indicating consistent behavior. A representative absorbance spectra of pure CpDs is shown in Figure 5b.

The luminescence excitation data for all the samples indicated the main absorption band (Soret band ≈ 400 nm and less intensive Q-bands around 500, 530, 600 and 660 nm). The luminescence spectrum maxima for all the samples were located around 670 nm and 720 nm. The peak positions in the spectra of the dyes incorporated in the polymer matrix were similar to ones obtained for the solution of the chlorin-e6 [95]. This data indicate that CpDs incorporated in the polymer matrix retain their luminescent properties and long-wavelength absorption band (≈660 nm), which is beneficial for photodynamic therapy (PDT) applications.

### 3.8. Singlet Oxygen Generation

Next, experiments using indocyanine green (ICG) dye as a singlet oxygen probe were conducted to evaluate singlet oxygen generation by PHB-CpD matrices. ICG is a well-known cyanine dye approved by the FDA for different applications [96]. The sensitivity of the ICG dye toward singlet oxygen allows its use as a probe for singlet oxygen detection [97]. Among the remarkable features of ICG is fluorescence in the NIR-region, water solubility and nontoxicity [97]. Singlet oxygen oxides ICG, which is accompanied by the destruction of the dye chromophore system and dye absorption spectrum change [98].

Upon photoexcitation (>400nm) of the PHB-mC_4_H_10_ matrix in the presence of ICG, the decrease of the dye absorption was observed (Figure 5c). Both the dye solution (ICG) and blank solution (ICG + PHB based matrix without CpDs (blank)) showed a decrease of ICG absorbance around 5% (Figure 5c, inset). At the same time, in the case of the ICG solution in the presence of the PHB-mC_4_H_10_ matrix, the absorption decrease was almost 40%. The same results for all samples of PHB-CpD matrices were observed.

The proposed photosensitizing mechanism includes the gradual extraction of chlorin from the polymer matrix of the sample and subsequent energy transfer from the chlorin molecule triplet state to molecular oxygen. However, one should not rule out the possibility of triplet–triplet energy transfer from the chlorin molecule triplet state to ICG and subsequent triplet–triplet energy transfer from the ICG triplet state to molecular oxygen. Since the concentration of oxygen in water (2.6 × 10^−4^ M) is around 100 times higher compared to the ICG concentration used (2.3 × 10^−6^ M), the role of this sensitizing pathway is considered to be insignificant. The obtained experimental data indicate the novel material exhibits photosensitizing properties and could potentially be implemented as light-induced singlet oxygen generators for PDT applications.

### 3.9. Cytotoxic Activity of CpDs

**Figure 6:**
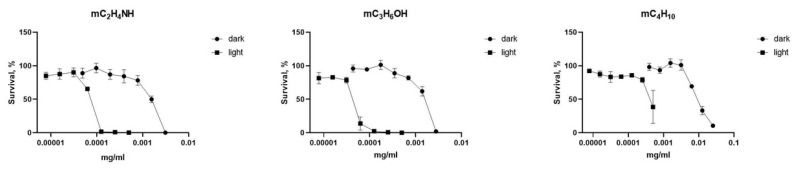
Survival of A431 cells after 72 h of incubation in the presence of photoactive substances without irradiation (dark) and after irradiation at 660 nm during 20 min with light intensity 1.7 J/cm^2^ (light).

The cytotoxicity of CpDs under light and dark conditions depends on the concentration of the PS [68]. Light excitation can significantly reduce the working dosages of PS. However, many chlorophyll derivatives exhibit strong cytotoxic activity against cancer cells even without light excitation [99]. To evaluate the optimal concentrations of effective use, all CpDs were predissolved in DMSO to ensure solubility and lack of visible crystals in the wells during the whole period of incubation observed. Figure 6 shows the survival of A431 cells after 72 h of incubation in the presence of photoactive substances without irradiation and after irradiation at 660 nm during 20 min.

**Figure 7:**
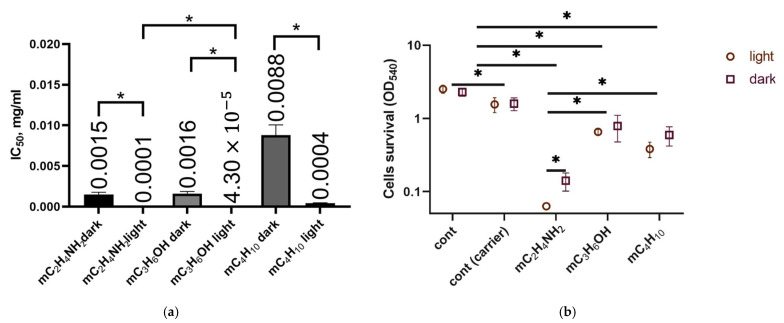
IC50 values of the photoactive substances without irradiation (dark) and irradiated at a wavelength of 660 nm for 20 min with light intensity 1.7 J/cm^2^ before incubation towards A431 cells after 72 h of incubation, * *p* < 0.05 (**a**). OD540 absorbance values of PHB-CpD matrices after MTT-test; cont—untreated control cells, * *p* < 0.05 (**b**).

The substances showed significant levels of photo-induced activity. Control cells, both irradiated and incubated in the dark, exhibited similar survival rates. The values of dark and photo-induced toxicity differ significantly (Figure 7).

mC_4_H_10_ revealed the lowest level of the dark-cytotoxicity (IC50 0.0088 mg/mL), but irradiation augmented the effect and reduced IC50 down to 0.0004 mg/mL. The dark-toxicity of the mC_2_H_4_NH and mC_3_H_6_OH was similar—0.0015 and 0.0016 mg/mL, respectively. mC_3_H_6_OH revealed the highest phototoxicity (IC50 0.000043 mg/mL), while mC_2_H_4_NH was slightly less active (0.0001 mg/mL), but the difference was significant (*p* < 0.05). The difference between dark and light-induced phototoxicity was significant for every substance—a 15–37-fold increment after irradiation was obsereved.

### 3.10. Cytotoxic Activity of PHB-CpD Matrices

The polymer matrix can significantly impact the operation of the PS by coordinating delivery and ensuring the gradual release of the substance [100]. Considering the high cytotoxic activity of CpDs, the study of the system based on polymer with CpDs is of great interest in comparison with pure PS. The substance-loaded matrices were cut into identical disks (1 cm diameter) to ensure results comparability among the samples.

Figure 7b shows the results of the MTT test of incubation in the presence of photoactive substances without irradiation and after irradiation at 660 nm for 20 min. In the case of pure PHB membranes (control samples), the lack of photo-induced toxicity was obsereved. Both samples demonstrated equal cell survival (Figure 7b). When comparing light-treated samples, the significantly enhanced cytotoxicity of substance-loaded samples was observed. But, in the case of mC_4_H_10_ and mC_3_H_6_OH, the difference was absent between light-treated samples and samples kept in the dark. This cytotoxicity absence was likely related to the hydrophobic nature of the mC_4_H_10_ and mC_3_H_6_OH substances and correlated with the cumulative release results. This assumption was also confirmed by the significant difference in dark and light-induced toxicity in the mC_2_H_4_NH case—fast release explains the highest activity, despite the average IC50 of the substance, where pure mC_3_H_6_OH demonstrated outstanding results. But, it is worth mentioning that these results are preliminary—many factors could influence outcomes, starting from the physico-chemical characteristics of fibers, spun material treatment, culture medium layer thickness, soaking time, release environment, pH, loading efficacy, etc. Considering these various factors, we can realize why we did not observe a 15–37-fold difference in the irradiated spun material case, which we detected during the MTT test of the substances.

**Figure 8:**
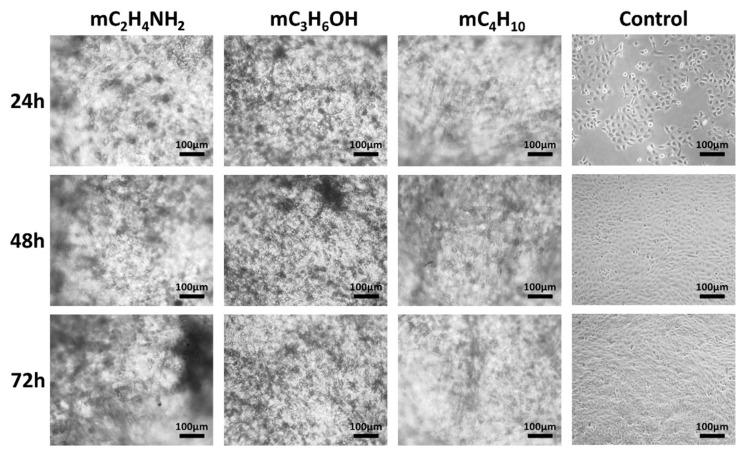
Images of the substance-loaded PHB membranes and A431 cells on the fibers 24, 48 and 72 h after seeding and 0, 24 and 48 h after irradiation.

A lack of obvious abnormalities during incubation was observed. Control cells had typical morphology and could be characterized by a normal growth rate; the cells seeded on the membrane surface could be clearly distinguished within the PHB membranes (Figure 8). It could be clearly observed that the spindle-shaped cells adhered to the surface of polymer fibers; in contrast to control cells (in the absence of membranes), the cells grew much slower, which could be noted visually, which was probably due to many factors—the spatial structure of the polymer, the limited possibility of cell division on fibers, limited intercellular interaction, the nature of the polymer, and the toxicity of the loaded substances. It should be noted that despite the limited toxicity of the substances loaded into the fibers, cell survival in the presence of the membranes was reduced. It follows from the data obtained that the proposed PHB-CpD matrices are effective not only at the time of irradiation but also even in dark conditions. Due to the highly developed biomimetic structure, controlled bioresorption without requirements to be removed from the body, the possibility of prolonged release of PS, and the generation of singlet oxygen over a long-time interval, the materials can be recommended for effective advanced therapy of carcinoma.

Several research groups have demonstrated the high biological activity of chlorophyll derivatives, including CpDs, which possess high PDT activity and are applied often as potent anticancer drugs. Along with excessive ROS generation, mitochondria failure and subsequent apoptosis were identified as the key players contributing to cancer eradication [101]. Moreover, Cho and colleagues demonstrated significant depression of the ERK pathway and reduced intracellular kinase activity in the process of ROS generation during PDT as well in the dark, explaining the high antiproliferative activity of CpDs. Thus, continuous release of CpDs during polymer amorphous phase destruction initiated by contact with cells and culture media was observed. CpD entrapment, low crystallinity (<60%), developed fiber surface, and the physico-chemical properties of a substance allow initiation of the release process quickly: the fastest release demonstrated the samples loaded with mC_2_H_4_NH. That was, in our opinion, the key factor ensuring high dark and light-induced anticancer activity even at low concentrations.

Summing up, the release rate is determined by the contact area of the polymer matrix with the environment and the degree of its development. In turn, the degree of surface development depends on the electrospinning conditions, which could be changed by introducing an additive. Differences in the curing rate and enthalpy of dissolution of additives determine the surface morphology and, consequently, the release profile.

### 3.11. Antimicrobial Test

The development of malignant skin neoplasms is associated with alterations in the dermal microbiome. These changes disrupt the balance between commensal and opportunistic bacteria. In particular, active overgrowth of *S. aureus* has been reported [102,103]. Its toxins induce oxidative stress, which damages the host cell’s DNA and impairs DNA repair mechanisms [104,105]. Persistent staphylococcal infections may also contribute to the formation of an immunosuppressive microenvironment that supports skin cancer progression [106]. Effective management of malignant skin neoplasms may therefore require approaches aimed at reducing bacterial load. The increasing prevalence of antibiotic-resistant *S. aureus* strains limits the effectiveness of conventional antibiotics [107,108,109]. Against this background, this study assessed both the antitumor and anti-staphylococcal activity of CpDs. The results obtained are shown in Table 5.

Unloaded PHB matrices showed no antibacterial activity against *S. aureus*. Bacterial growth was comparable to the control. By contrast, CpD-loaded matrices showed a concentration-dependent effect, with reduced efficacy at higher bacterial loads. PHB-mC_3_H_6_OH inhibited growth only at 10^4^ CFU/mL, both under irradiation and in the dark. Irradiated PHB-mC_4_H_10_ and PHB-mC_2_H_4_NH_2_ inhibited growth across all tested concentrations, whereas in the absence of irradiation activity it was limited to ≤10^4^ CFU/mL. Free mC_3_H_6_OH showed the highest antibacterial activity. Its effect exceeded that of other derivatives by 24–29% in the dark and by 29–30% under irradiation. Interestingly, free compounds were more active than polymer-bound forms, likely due to the gradual release of CpDs from the polymeric matrix. Irradiation enhanced antibacterial activity in all compounds. The inhibitory effect of free mC_4_H_10_, mC_2_H_4_NH_2_, and mC_3_H_6_OH increased by 20%, 26%, and 29%, respectively. The corresponding increases for polymer-bound forms were 26%, 17%, and 33%.

Pheophorbide is considered as a light-triggered molecule that induces oxidative stress in microbial cells by generating singlet oxygen [110,111]. Nonetheless, B.Ch.L. Chan et al. found a light-independent effect of pheophorbide, presumably caused by its intrinsic toxicity [111]. In addition, M. Kraatz et al. reported its ability to inhibit bacterial efflux pumps even in the dark [112]. These findings suggest that similar mechanisms may underlie the activity of the derivatives evaluated in this study. However, this assumption requires further detailed investigation.

### 3.12. Bacterial Cell SEM Results

**Figure 9:**
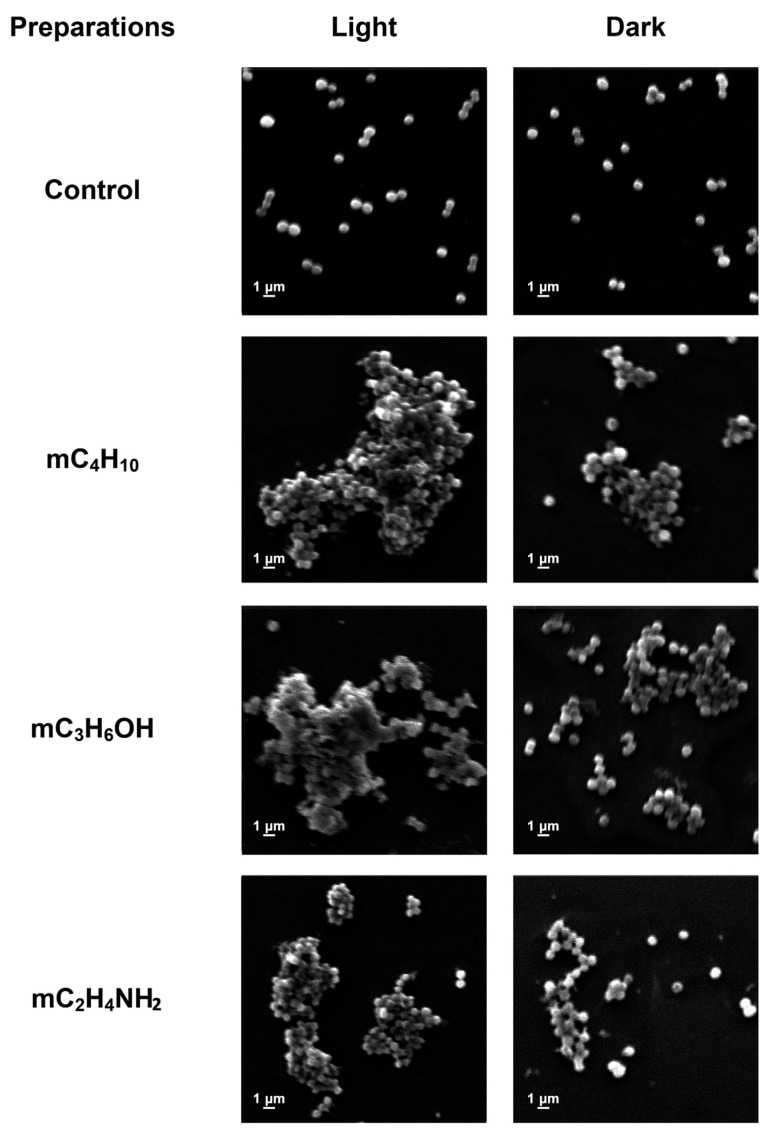
SEM images of *S. aureus* cells, incubated with irradiated and non-irradiated CpDs (bar, 1 μm).

The morphological changes in *S. aureus* cells induced by CpDs under irradiation (16.2 J/cm^2^ for 25 min) and in the dark were assessed by SEM (Figure 9).

The control images showed clear edges as well as a characteristic size and shape of bacterial cells. *S. aureus* cells exposed to CpDs deformed and aggregated, forming conglomerates. Treatment with the same concentration of irradiated derivates resulted in lysis of the cell walls and the complete destruction of bacteria. Hence, destructive processes were more pronounced, which is consistent with our results for the IZD analysis. D.B. DuBois et al. [113], X. Nie et al. [114], and A. Mensah et al. [115] noted a similar effect of irradiation on the bacterial cell wall.

## 4. Conclusions

A new type of biomimetic materials encapsulated with chlorophyll derivatives for PDT based on PHB fibers were successfully fabricated by the ES method. These PHB-CpD matrices showed a potential for controlled prolonged delivery of photodynamic therapeutic reagents to targeted regions. CpDs showed a significant impact on the efficiency of the ES process even in case of low concentrations of therapeutic substance (less than 1%). Thus, a 40% increase in electrical conductivity for all additives made it possible to compensate the increase in viscosity and led to a general decrease in average diameters of fibers by 35–40% and to the decrease in the spread of values. Moreover, the CpDs had a multidirectional effect on the formation of the microrelief of the surface due to the effect on the evaporation rate of the solvent during the curing of the fibers. This, combined with the peculiarities of the organization of the crystalline structure and the interfibrillar space, led to significant changes in the nature of release of CpDs from the PHB matrix. So, within 22 days, the release of 32% of the substance for PHB-mC_2_H_4_NH_2_ was established, 9% for PHB-mC_4_H_10_ and 2% for PHB-mC_3_H_6_OH. Our findings indicate also that the loading of CpDs into the PHB matrix not only improves the morphological characteristics of the fibers, reducing average diameter from 3.5 µm to 2.1 µm and increasing porosity from 80% to 90%, but also enhances the material’s hydrophilicity, achieving a 10% increase under various conditions, which is crucial for optimizing drug delivery systems.

It was shown that PHB-CpD matrices could generate singlet oxygen for extended periods. Moreover, luminescence spectrum maxima for PHB-CpDs were located around 670 nm and 720 nm, which is beneficial for deeper tissue penetration in PDT.

Irradiated CpDs in free and polymeric forms showed activity against *S. aureus*, which is a common bacterium that infects skin cancer areas. The developed materials have the potential to be used as a skin-interfaced multifunctional dressing that integrates antitumor and antimicrobial effects. Pure CpDs demonstrated clear PDT activity, revealing a 15–37-fold IC50 increment after irradiation; mC_3_H_6_OH showed the highest phototoxicity (IC50 43 ng/mL) towards carcinoma cells. After loading into the PHB matrix, we observed notable changes in CpD activity: while pure PHB membranes showed a lack of photo-induced toxicity, the mC_2_H_4_NH_2_-loaded matrix demonstrated the highest level of light-induced toxicity; mC_4_H_10_ and mC_3_H_6_OH loading showed a non-significant difference between control and light-treated samples. We explain this difference among CpD-loaded samples with release profiles. These variations in action modes can be exploited for rapid or prolonged treatment regimens.

## Figures and Tables

**Table 1:** Advantages and disadvantages of polymer-based CpD carriers for PDT.

Type of Carrier	Polymers	Advantages	Disadvantages	References
Polymeric Micelles	Chitosan Phospholipids Poly(ethylene glycol)-poly(d,l-lactide)	High loading of PS; Possibility of stimuli-responsive drug release	Fast degradation and possibility of early burst release; Rapid clearance from the body	[25,26,27]
Polymeric lyposomes	Poly(ethylene glycol) Polyphosphoester Polyvinylpyrrolidone	Effective encapsulation and improved pharmacokinetics of photosensitizers	Limited physicochemical stability under physiological conditions	[28,29,30]
Conjugated Polymer Nanoparticles	Poly(dopamine) Poly(amidoamine) Poly(ethylene glycol) Chitosan Gelatin	High absorption cross-section; Excellent photostability; High ROS generation	Laborious synthesis; High production cost; Rapid clearance from the body; Uncontrolled degradation rate in the body	[31,32,33,34,35]
Hydrogels and Nanoemulsions	Gellan Gum Alginate Carboxymethyl Cellulose	Localized, high-precision delivery; Biocompatibility	Poor deep tumor penetration; Limited effectiveness in certain applications	[36,37,38]
Porous Polymers	Cellulose Silk Fibroin Polyhydroxybutyrate	Photostability; High biocompatibility; High ROS generation	Aggregation; Slow biodegradation	[39,40]

**Table 2:** ES conditions of PHB-CpD matrix fabrication.

Sample	Voltage, kV	Electrical Conductivity, µS/cm	Viscosity, Pa s	Average Diameter, µm
PHB	18	10	1.0	3.5
PHB-mC_4_H_10_	16	14	1.2	2.6
PHB-mC_3_H_6_OH	16	14	1.2	2.1
PHB-mC_2_H_4_NH_2_	16	14	1.2	2.2

**Table 3:** Thermal properties of PHB-CpD matrices.

	1 Heating			2 Heating		
Sample	Melting Temperature, °C	Melting Enthalpy, J/g	Crystallinity Degree, %	Melting Temperature, °C	Melting Enthalpy, J/g	Crystallinity Degree, %
PHB	174	93	64	170	84	58
PHB-mC_4_H_10_	176	80	54	174	79	54
PHB-mC_3_H_6_OH	175	77	53	172	76	52
PHB-mC_2_H_4_NH_2_	176	82	56	174	79	54

**Table 4:** Mechanical properties of the PHB-CpD matrices (* *p* < 0.001; ** *p* < 0.0001).

Sample	Tensile Strength, N/mm^2^ (±S.D., n = 10)	Elongation at Break, % (±S.D., n = 10)	Young’s Modulus, MPa (±S.D., n = 10)
PHB	0.90 ± 0.02	4.9 ± 0.2	38 ± 10
PHB-mC_4_H_10_	1.20 ± 0.03 *	3.9 ± 0.2 **	698 ± 30 *
PHB-mC_3_H_6_OH	0.10 ± 0.02 **	2.0 ± 0.2 **	74 ± 4 **
PHB-mC_2_H_4_NH_2_	0.50 ± 0.02 **	4.0 ± 0.3 **	254 ± 26 *

**Table 5:** Inhibition effect of CpDs and PHB-CpD matrices on *S. aureus*.

	Inhibition Zone Diameter (IZD), mm					
	Irradiation			No Irradiation		
Samples	10^4^ CFU/mL	10^5^ CFU/mL	10^6^ CFU/mL	10^4^ CFU/mL	10^5^ CFU/mL	10^6^ CFU/mL
PHB	0	0	0	0	0	0
mC_4_H_10_	30 ± 2	26 ± 2	24 ± 1	25 ± 2	0	0
PHB-mC_4_H_10_	24 ± 2	21 ± 1	20 ± 2	19 ± 1	0	0
mC_3_H_6_OH	39 ± 3	22 ± 2	17 ± 1	31 ± 2	16 ± 1	0
PHB-mC_3_H_6_OH	28 ± 2	0	0	24 ± 2	0	0
mC_2_H_4_NH_2_	31 ± 3	28 ± 2	22 ± 1	24 ± 1	0	0
PHB-mC_2_H_4_NH_2_	28 ± 2	25 ± 3	20 ± 2	21 ± 1	0	0

## Data Availability

The original contributions presented in this study are included in the article. Further inquiries can be directed to the corresponding author.
